# Low frequency detection in clinical multispectral optoacoustic tomography

**DOI:** 10.1016/j.pacs.2025.100785

**Published:** 2025-11-17

**Authors:** Maximilian Bader, Antonia Longo, Dominik Jüstel, Vasilis Ntziachristos

**Affiliations:** a Chair of Biological Imaging, Central Institute for Translational Cancer Research (TranslaTUM), School of Medicine and Health & School of Computation, Information and Technology, Technical University of Munich, Munich, Germany; b Institute of Biological and Medical Imaging, Bioengineering Center, Helmholtz Zentrum München, Neuherberg, Germany; c iThera Medical GmbH, Munich, Germany; d Institute of Computational Biology, Computational Health Center, Helmholtz Zentrum München, Neuherberg, Germany; e Institute of Electronic Structure and Laser (IESL), Foundation for Research and Technology Hellas (FORTH), Heraklion, Greece; f Munich Institute of Biomedical Engineering (MIBE), Technical University of Munich, Garching b. München, Germany

**Keywords:** system characterization, frequency calibration, impulse response, frequency response, broadband acoustic waves

## Abstract

Frequency response characterization of optoacoustic (photoacoustic) detectors is critical because the signals are broadband, and the center frequency relates to the absorber size. We were particularly interested in studying the sub-1 megahertz response, which enables imaging of low spatial frequencies associated with resolving organs up to centimeter size. State-of-the-art characterization methods fail to measure this kilohertz frequency response reliably, leading to an incomplete understanding of the largest structures captured in an optoacoustic image. Herein, we developed an experimental arrangement to identify the lowest measurable frequency of an optoacoustic detector. We observe that a common optoacoustic detector with a 3.4 megahertz center frequency and 72 % 3 dB bandwidth can capture signals as low as 75 kilohertz. Given insufficient characterization methods, we also investigate artifacts triggered by image reconstruction with an erroneous kilohertz frequency response. Collectively, our work discusses the impact of incorporating low frequencies on optoacoustic image fidelity.

## 1 Introduction

Optoacoustic (OptA) imaging, also termed photoacoustic imaging, utilizes light of transient energy to excite ultrasound waves within tissue, due to thermoelastic expansion. Handheld OptA imaging, in particular Multispectral Optoacoustic Tomography (MSOT Acuity Echo CE® by iThera Medical GmbH, Munich, Germany) and Optoacoustic Ultrasound (Imagio® by Seno Medical Instruments Inc., San Antonio, USA), has been recently identified as the modality primarily employed in human optoacoustic studies [1], [2].

Common implementations geared for handheld clinical operation utilize illumination with short light pulses in the tens of nanoseconds range, that excite ultrasound waves due to thermoelastic expansion. These waves are captured using transducers with central frequencies in the Megahertz (MHz) range [3]. Assuming that these light pulses employed for illumination act as Dirac delta excitation, the frequency content of the resulting ultrasound waves is broadband and depends on the size distribution of tissue absorbers. Generally, low frequencies are primarily generated by large structures and high frequencies by smaller structures [3], [4], [5], [6]. Image reconstruction based on back-projection approaches preferentially renders the high frequencies and, therefore, primarily reconstructs tissue vasculature, i.e., small-sized light-absorbing structures [7]. Large structures will not be visualized completely in the images, but only boundaries may appear in the image, i.e., a spatially high pass filtered image of the large structures [8]. Nevertheless, it is well recognized that the detection and processing of low frequencies is beneficial to image fidelity as it allows the reconstruction of more complete representation of the imaged object, including imaging of larger organs and interfaces [7], [9]. Moreover, since high frequencies are attenuated at a higher rate than lower frequencies, as a function of ultrasound propagation distance, the frequency content of the OptA signals detected also relates to the depth that can be interrogated.

Given the generation of such broadband responses, the frequency content available to the optoacoustic reconstruction relates to the bandwidth of the ultrasound transducer (UT) and acquisition electronics employed. Therefore, characterization of the detector bandwidth, i.e., measurement of its frequency response, is useful for understanding and designing the operational characteristics of an optoacoustic system [8], [10]. This is in contrast to ultrasonography needs, whereby images are typically generated at a narrow bandwidth around a single frequency [11]. Therefore, detector characterization does not commonly focus on bandwidth related measurements, especially in relation to characterizing the lowest frequency measurable by a detector.

Characterization of UTs for use in OptA has been achieved so far by illuminating absorbing microspheres [12], [13]. However, this method is not suitable for determining the sub-1 MHz UT’s frequency response at frequencies smaller than 1 MHz due to the low OptA signal amplitude generated by microspheres and possible electronic noise interference [14], [15]. Another technique, termed substitution source measurement, commonly applied to characterize acoustic output in medical ultrasound, uses a second, calibrated UT to emit ultrasound waves with known pressure amplitude at the sonography device [16]. Substitution source measurements have been applied to measure the peak sensitivity of an OptA imaging device at its center frequency of 1 MHz [17]. However, because the secondary, transmitting ultrasound transducers (Tx-UTs) in substitution source measurements have center frequencies in the MHz range, typically matched to the central frequency of the ultrasound element characterized, it is not possible to measure the lowest detectable frequency in the Kilohertz (kHz) range.

In this work we aimed to determine the lowest frequency detected by a commercial clinical macroscopic OptA imaging device for the first time. To achieve this goal, we developed an experimental arrangement that implemented the substitution source method using secondary Tx-UTs with bandwidths covering the frequency range from 25 kHz to 1 MHz. We experimentally measured the signal detected by a clinical handheld OptA system and determined the signal to noise ratio (SNR) achieved in the different frequencies measured. To provide a reference, we also compare the SNR determined with our proposed method with the state-of-the-art UT characterization employing laser-illuminated microspheres. To elucidate the effect of the low frequencies identified by our measurements, we further performed reconstructions using simulated OptA signals from tissue-mimicking geometries, i.e., in-silico phantoms. The reconstructions utilized electrical impulse responses that over- and underestimated the low-frequency content, so as to generate a reference on the artifacts that are associated with the low frequency content in the data. We discuss our findings and the implications of low frequencies on the quality on optoacoustic images which should serve operators as guidance to judge macroscopic OptA image fidelity better.

## 2 Methods

### 2.1 Theory – wave propagation and detection in optoacoustic imaging

The acoustic wave equation describes the pressure p at spatial location $x\in R^{3}$ and time t∈R in a medium with speed of sound c for a given acoustic source S(x,t) as shown below:

(1) $$\frac{\partial^{2}}{\partial t^{2}}p\left(x,t\right)-c^{2}\Delta p\left(x,t\right)=S(x,t)$$

c is assumed to be constant. Acoustic attenuation and dispersion can be neglected in water (see Supplementary Fig. A.2a,b) [13], [18], [19]. For OptA signals, we consider that acoustic waves are generated by short laser pulses in the regime of thermal and stress confinement. The incident light is absorbed in tissue and converted to acoustic energy via the optoacoustic effect, inducing an initial pressure distribution $p_{0}\left(x\right)≔p\left(x,0\right)$ with negligible initial velocity. The resulting source term in Eq. (1) is $S^{\mathrm{OptA}}\left(x,t\right)=\delta^{\prime }\left(t\right)p_{0}\left(x\right)$, where $\delta^{\prime }$ denotes the time-derivative of the Dirac-delta function [20]. When generating pressure with a focused UT, we simplify wave generation by assuming a virtual transmitter in the UT focus point (focal spot size of UT approximately equals the resolution of the Rx-UT E), which induces a temporal acoustic pulse w. This pulse may be introduced into Eq. (1) as source term $S^{\mathrm{UT}}\left(x,t\right)=w\left(t\right)\delta (x-x\prime )$, where x′ denotes the virtual UT location [21], [22]. Given the solution of Eq. (1) for an OptA pressure or a pressure generated with a focused UT, an receiving ultrasound transducer (Rx-UT) E records the acoustic signal $s_{p}^{E}$ over time t∈R:

(2) $$s_{p}^{E}\left(t\right)≔P*\mathrm{SI}R_{x^{\prime }}^{E}*\mathrm{EI}R^{E}\left(t\right)$$

where $\mathrm{SI}R_{x\prime }^{E}$ denotes the spatial impulse response (SIR_x_) of E for a source located at x′, $\mathrm{EI}R^{E}$ is the electrical impulse response (EIR) of E, P is the acoustic excitation shape and * is the temporal convolution operator [20]. For OptA excitation, we assume a radially symmetric $p_{0}$ with profile $p_{0}^{\mathrm{profile}}\left(r\right)≔p_{0}\left(\left|x\right|\right)$ for r∈R. This $p_{0}$ triggers an acoustic wave which results in an excitation $P(t)=N_{p_{0}}\left(t\right)=-c^{2}tp_{o}^{\mathrm{profile}}(\mathrm{ct})$, the so-called generalized N-shape $N_{p_{0}}\;$[23]. For wave generation with a focused UT, the excitation is given by P(t)=w(t). The SIR_x_ describes the pressure detected by a UT over time t given a Dirac-delta-like acoustic source located at location x [24], [25] while the EIR models the conversion of pressure to electric signal in the transducer and the acquisition electronics. The EIR is the time-domain equivalent of the frequency response and hence, encapsulates the limited bandwidth of the UT [14], [15]. A clinical OptA imaging device consists of a UT array ${\left\{E_{k}\right\}}_{k=1}^{K}$ with K elements and provides digital signals sampled with sampling frequency $f_{s}$. The set of signals acquired by all transducers at time samples ${\left\{t_{j}\right\}}_{j=1}^{T}$ is called sinogram $S_{j,k}≔s_{S}^{E_{k}}\left(t_{j}\right),\;j=1,\ldots ,T$.

### 2.2 Frequency characterization and signal-to-noise ratio measurements for a clinical optoacoustic imaging device

**Fig. 1:**
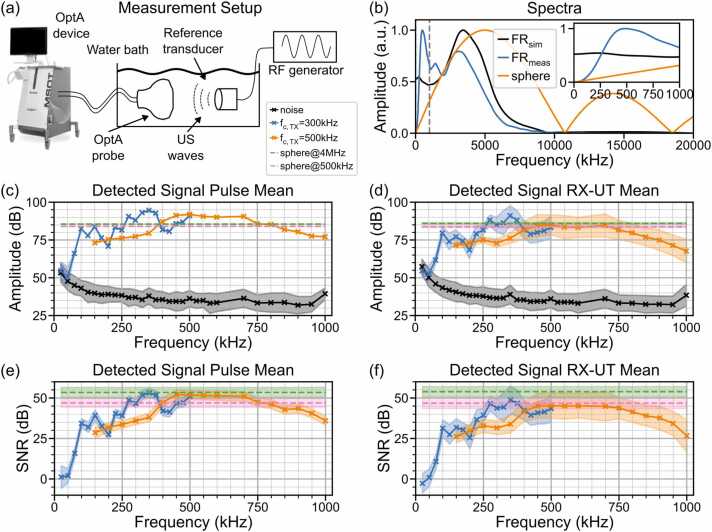
Measured low-frequency signal amplitude and signal-to-noise ratio (SNR) of a clinical macroscopic optoacoustic (OptA) imaging device for frequencies between 25 kHz and 1 MHz. (a) Photo and schematic of a low-frequency measurement setup using the probe of a clinical OptA imaging device (MSOT Acuity Echo CE®, iThera Medical GmbH, Munich) and a reference transmitting ultrasound transducer (Tx-UT). (b) Simulated (FR_sim_, black line) and measured (FR_meas_, blue line) frequency response (FR) of the OptA imaging device shown in (a) and the spectrum of the optoacoustic signal generated by a sphere (orange, diameter: 200 µm) over a frequency range of 0–20 MHz. The inset displays the sub-1 MHz spectra between the y-axis and the gray line. The simulated frequency response was obtained using a KLM model, while the measured frequency response was derived from optoacoustic (OptA) microsphere measurements (diameter: 200 µm, bandpass filter: 50kHz-12MHz). (c,d) Mean and standard deviation (std) of the detected signal amplitude across a frequency range from 25 kHz to 1 MHz, for a single receiving ultrasound transducer (RX-UT) averaged over 100 pulses (c) and for a single pulse averaged over 256 RX-UTs (d) for Tx-UTs with center frequencies of 300 kHz (blue) and 500 kHz (orange). Noise is shown in black. For comparison, dashed lines show the acoustic signal amplitude generated by a laser-illuminated microsphere at frequency f=4MHz (green) and at f=500kHz (pink). (e,f) Mean and std of the detected SNR for a single RX-UT averaged over 100 pulses (e) and for a single pulse averaged over 256 RX-UTs (f) for Tx-UTs with center frequencies of 300 kHz (blue) and 500 kHz (orange). For comparison, dashed lines show the SNRs of the laser-illuminated microsphere at frequency f=4MHz (green) and at f=500kHz (pink). RF – radio frequency.

Fig. 1a depicts the measurement setup to probe the sub-1 MHz sensitivity of a clinical OptA imaging device (MSOT Acuity Echo CE®, iThera Medical GmbH, Munich, Germany, 256 UT array, array radius: 4 cm, angular coverage: 125°, elevational focus radius of a single transducer: 4 cm, transducer center frequency: 3.4 MHz, 3 dB bandwidth: 72 %, 6 dB bandwidth: 113 %, sampling frequency: 40 MHz, recorded samples per cycle: 2030, pulse repetition rate: 25 Hz). Following the substitution source measurement technique [16], we submersed the device’s handheld probe in a water bath and placed two reference Tx-UTs with 300 kHz and 500 kHz center frequencies (300 kHz: 10 mm element size, 6 mm spherical focus; 500 kHz: 9 mm element size, 4 mm spherical focus; Sofranel, Sartrouville, France) facing the OptA probe. Both the Tx-UT and the probe were fixed inside the water bath at a distance of 7.9 cm from the UT array facing each other using optical posts. The Tx-UT and probe’s detectors were first aligned in elevational direction (out-of-plane) by maximizing the detected signal amplitude. In a second step, the Tx-UT was aligned with the lateral center of the probe’s UTs. We matched the signal peak arrival times at the two outermost UTs in the array which corresponded to a perfectly horizontal beam pattern throughout the live image provided by the imaging device. In order to measure the signal recorded by the OptA imaging device for frequencies below 1 MHz, the Tx-UT was excited with a single-frequency sinus signal $v\left(t\right)=V_{\mathrm{pp}}\sin(2\pi f_{\mathrm{TX}}t)$ using a radio frequency (RF) waveform generator (33500B Series Keysight Technologies Inc., Santa Rosa, USA). The OptA imaging device recorded the resulting ultrasound wave for a duration of 10 s, corresponding to ∼250 acquisition cycles. For both Tx-UTs manual frequency sweeps were conducted to cover the frequency band between 25 kHz and 1 MHz (300 kHz: $V_{\mathrm{pp}}=2.5\;V$, $f_{\mathrm{TX}}\in \left\{i\cdot 25\mathrm{kHz}\;\right|\;1\leq i\leq 20\}$, $∆f_{\mathrm{TX}}=25\mathrm{kHz}$, 500 kHz: $V_{\mathrm{pp}}=2\;V$, $f_{\mathrm{TX}}\in \left\{i\cdot 50\mathrm{kHz}\;\right|\;3\leq i\leq 20,\;i\neq 12\}$, $∆f_{\mathrm{TX}}=50\mathrm{kHz}$).

To record the noise, we switched the RF waveform generator off and acquired data without transmitting any acoustic signal. During all measurements with a Tx-UT, the OptA imaging device’s laser was deactivated to avoid OptA signal interference from the membrane. We also recorded the acoustic signal of a laser-pulse illuminated microsphere (diameter: 200 µm, black paramagnetic polyethylene microspheres, Cospheric LLC, Somis, USA) at nine separate locations in the focal plane of the OptA probe’s transducer array [11], [12]. During the microsphere measurement, the OptA probe was disassembled so that it consisted only of the transducer array and data acquisition unit in order to inhibit signal alterations by other probe components like the probe membrane. Optical wavelength and laser energy were optimized to obtain optimal signal amplitude during the measurement. The signal data were automatically averaged across 100 acquisition cycles to improve the measurement SNR. We derived the OptA imaging device’s EIR from the microsphere measurements using the synthetic total impulse response (sTIR) approach [12]. As a reference served the simulated EIR obtained using an equivalent-circuit model (KLM) for the UTs and detection electronics of the MSOT Acuity Echo CE® device (iThera Medical GmbH, Munich, Germany) [26].

To compare the signal and noise amplitude, we computed the demeaned amplitude spectrum of each sinogram ${\hat{S}}_{j,k}^{\mu =0}$ at a given discrete acoustic frequency j (f=j⋅∆f) using the equation below:

(3) $${\hat{S}}_{i,k}^{\mu =0}≔F\left[s_{S,l}^{E_{k}}-\mu_{s,k}\right]\left(j\right),$$

where $F\left[f\right]\left(j\right)≔\hat{f}\left(j\right)≔\sum_{n=0}^{N-1}f_{n}\exp(-i2\pi \frac{j}{N}n)$ denotes the discrete Fourier transform, N denotes the discrete frequency resolution, and $\mu_{s,k}=\frac{1}{T}\sum_{j=1}^{T}s_{S,l}^{E_{k}}(t_{j})$ is the signal mean of transducer $E_{k}$. We selected a frequency resolution of ∆f=25kHz, resulting in a fast Fourier transform (FFT) length of $N=\frac{f_{s}}{∆f}+1=\frac{40\mathrm{MHz}}{25\mathrm{kHz}}+1=1601$. For the Tx-UTs, we computed the amplitude spectrum for all $f_{\mathrm{TX}}$. As a reference, we computed the amplitude spectrum of the microsphere located in the focal point of the UT array at f∈{500kHz,4MHz}, because at both frequencies the microsphere signal is sufficiently large to differentiate it from noise. We determined amplitude spectra both dependent and independent of the acoustic pulse shape of the microsphere and the Tx-UT frequency responses, deconvolving the source frequency response from the signal spectrum according to Eq. (2). For the Tx-UT frequency response, we used the square-root of the pulse-echo frequency response provided by the UT manufacturer. For the frequency response of the microsphere, we computed the normalized spectrum of the acoustic signal generated by a liquid sphere at constant speed of sound c=1510m/s [4]. In both cases, we neglected the SIR_x_ effect, because the amplitude variance between 0 and 1 MHz is negligible for a source in the far-field (see Supplementary Fig. A.2c,d) and the SIR_x_ for a sphere located in the transducer array focus is a Dirac-delta with a constant frequency response across all frequencies. For verification, we simulated the SIR_x_ for virtual transducer locations in the far-field using the Field II framework [27], [28].

Based on the amplitude spectra of the signal and noise sinograms, we determined the signal to noise ratio $\mathrm{SN}R_{i,k}$ by:

(4) $$\mathrm{SN}R_{i,k}=W_{i,k}^{\mathrm{sig}}/W_{i,k}^{\mathrm{noise}}$$

where $W_{i,k}^{\mathrm{type}}=\sum_{j=i-1}^{i+1}{∆f\left|{\hat{S}}_{j,k}^{\mu =0}\right|}^{2},\mathrm{type}\in \{\mathrm{sig},\mathrm{noise}\}$ is the energy of either signal or noise in the frequency band $f\in [f_{\mathrm{TX}}-25\mathrm{kHz},f_{\mathrm{TX}}+25\mathrm{kHz}]$. For both the amplitude and SNR of the UT data, we report the mean and std on a decibel scale across all Rx-UTs of the array and a single acquisition cycle and as well as across 100 acquisition cycles and a single Rx-UT in the center of the array.

Using an approximation of the lower −3 dB cutoff frequency $f_{\mathrm{lower},1}$ of the OptA signal generated by a sphere with diameter $D_{s}$, we determined the largest sphere visualizable without spatial lowpass filtering [20], [29]:

(5) $$D_{s}\approx 0.32\frac{c}{f_{\mathrm{lower},1}}.$$

Analog, we determined the maximum thickness $d_{l}$ of a layer which can still be resolved given a −3 dB cutoff frequency $f_{\mathrm{lower},1}$ of the OptA signal [4]:

(6) $$d_{s}\approx 0.4429\frac{c}{\pi f_{\mathrm{lower},1}}.$$

To estimate the maximum size of a cylinder whose OptA signal can still be resolved given a lower −3 dB cutoff frequency $f_{\mathrm{lower},1}$, we calculated the OptA signal numerically using the given analytic solution [30].

### 2.3 Model-based image reconstruction for a clinical optoacoustic imaging device

To perform OptA image reconstruction, we employed the model defined in Eq. (2) in a model-based reconstruction framework [13], [23], [31]. We discretized the initial pressure distribution $p_{0}$ in a shift-invariant space and selected a regular lattice $L_{a}$ with spacing a and a radially symmetric Gaussian generator $G_{\sigma }\left(x\right)≔\frac{1}{\sqrt{{\left(2\pi \right)}^{3}\sigma^{2}}}e^{-{\left|x\right|}^{2}/2\sigma^{2}}$ with σ=a/2. Following Eq. (2) the resulting discretized model can be written as:

(7) $$s_{p_{0}}^{E_{k}}\left(t_{j}\right)≔\sum_{l\in \Omega {(L}_{a})}p_{0,l}\cdot N_{G_{\sigma }}*\mathrm{SI}R_{l}^{E_{k}}*\mathrm{EI}R^{E_{k}}\;j=1,\ldots ,T,\;k=1,\ldots ,K$$

where $N_{G_{\sigma }}$ denotes the N-Shape for a Gaussian initial pressure profile $p_{o}^{\mathrm{profile}}(r)=G_{\sigma }(x)$, $p_{0,l}$ is the initial pressure projected into the shift-invariant space and $\Omega (L_{a})$ is a finite subset Ω of the regular lattice $L_{a}\;$[23]. We then performed image reconstruction with variational methods, solving the optimization problem:

(8) $$p_{0}^{\mathrm{rec}}≔\arg\underset{p_{0}\geq 0}{\min}{\left\|\mathrm{Sys}\left[p_{0}\right]-S^{\mathrm{meas}}\right\|}_{2}^{2}\;,$$

where $\mathrm{Sys}:\;p_{0}\to S$ is the linear operator mapping initial pressure to sinograms and $S^{\mathrm{meas}}$ is the measured sinogram. We regularized the optimization problem by early stopping.

To investigate artifacts triggered by imprecise low-frequency characterization, we assumed that a numerical tissue-mimicking phantom was imaged by the same clinical OptA imaging device used for the SNR measurements (see Methods 2.2 for detailed specifications). We generated a stochastic, numerical breast tissue phantom using a stochastic image generator, which provided optical absorption values for wavelengths between 680 and 1130 nm with a step size of 10 nm [32]. We then simulated fluence employing a custom Monte-Carlo light modeling algorithm and extracted a single cross-sectional slice from the volume and cropped it to a two-dimensional field of view [x,z] (x∈[−2cm,2cm], z∈[−2cm,2cm]) with a lattice spacing, i.e., resolution, of a=100µm.

**Fig. 2:**
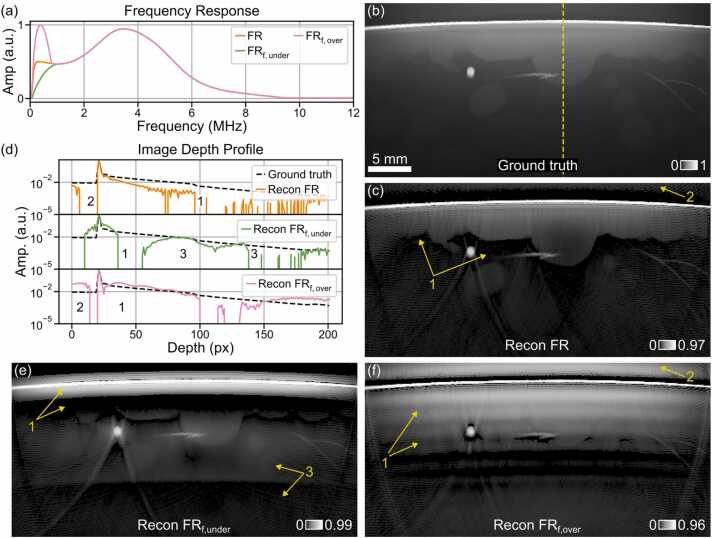
Numerical phantom experiment to qualitatively investigate the effects of imprecise transducer frequency response characterization for sub-1 MHz frequencies. (a) Simulated frequency response (FR) of a clinical macroscopic optoacoustic (OptA) imaging device with true (orange), over- (pink), and underestimated (green) amplitude for low frequencies (<1 MHz). (b) A numerical breast phantom at 930 nm. The yellow dashed line indicates the location of the image depth profiles displayed in (b), and the numbers correspond to those displayed in (b,c,e,f). (c) An image of the breast tissue phantom reconstructed with the true frequency response shows only the first lipid layer (arrow 1) and no bulk further below. Additionally, bulk contrast directly above the skin is reduced (arrow 2). (d) Depth profiles across all images (b,c,e,f) at the same lateral position indicated by the yellow dashed line in (b). (e) Image of the numerical phantom reconstructed with a frequency response which underestimates the low-frequency amplitude (FR_f,under_) with a considerably shortened and high-contrast entry layer (arrow 1) and a layer below the final layer which ends abruptly (arrow 3). (f) Image of the numerical phantom reconstructed with a frequency response overestimating the low-frequency amplitude (FR_f,over_) showing artificially enhanced layers (arrow 1) and a layer above the first bulk layer (arrow 2). Abbreviations: Amp. – amplitude, px – pixel.

To obtain the measured sinograms $S^{\mathrm{meas}}$, we applied the system model Sys to the initial pressure distribution of the phantom and applied a Butterworth bandpass signal filter (2nd order high-pass with cutoff $f_{3\mathrm{dB}}=100\mathrm{kHz}$, 8th order low-pass with cutoff $f_{3\mathrm{dB}}=18\mathrm{MHz}$). We simulated the SIR_x_ convolved with the Gaussian N-shape using the spatial pulse response method and solved the integral with the trapezoidal rule of integration [23]. As the ground truth EIR for the simulation, we employed a simulated EIR of the MSOT Acuity Echo CE® transducer (see Methods 2.2 for derivation) [18]. We then reconstructed the image with frequency responses of approximately 2- and ½-fold magnitude compared to the ground truth frequency response at f=400kHz. For the frequency response with an overestimated sensitivity (FR_f,over_), we scaled the EIR magnitude with an absolute sinus function between 0 and π, peaking at 400 kHz. The magnitude of the frequency response with underestimated sensitivity (FR_f,under_) was scaled with an absolute sinus function between 0 and π/2, peaking at 1 MHz. The true frequency response and the frequency responses with over- and underestimated low-frequency amplitude are plotted in Fig. 2a. To solve the optimization problem in Eq. (7), we used an iterative non-negative least-squares solver that was stopped early after 50 outer and two inner iterations.

For visualization, we processed all images with an image viewer specialized for MSOT (Image Viewer MK2) [33] applying gamma correction, local contrast enhancement, and sigmoid normalization to visualize the extensive dynamic range in the OptA images. In order to assess the image quality quantitatively, we computed the mean squared error $\mathrm{MS}E_{\mathrm{ref}}$ and structural similarity index $\mathrm{SSI}M_{\mathrm{ref}}$ [34]. We employed both the ground truth (GT) image and the reconstruction with true frequency response (Recon FR) as reference (ref) images for both metrics.

## 3 Results

### 3.1 Probing the sub-1 megahertz sensitivity

Fig. 1 shows the experimental arrangement that enabled substitution source measurements for frequencies between 25 kHz and 1 MHz. The arrangement probed the ultrasound element of a clinical OptA imaging device (MSOT Acuity Echo CE®, iThera Medical GmbH, Munich, Germany) and comprised a reference Tx-UT and the OptA system detector, positioned opposite each other in a water bath. The Tx-UT is driven with mono-frequency sinusoidal signals using an RF waveform generator, and the resulting ultrasound wave is detected with the Rx-UTs in the OptA probe. We used two separate Tx-UTs with a center frequency of 300 kHz and 500 kHz, respectively, to cover the sub-1 MHz sensitivity of the OptA imaging device (see Methods 2.2 and Supplementary Fig. A.1a).

The need for the proposed experimental arrangement to measure the kHz frequency response is highlighted in Fig. 1b. There is a clear mismatch between the expected, simulated (black), and the measured frequency response (blue) for frequencies below 1 MHz. The simulated frequency response was obtained using an equivalent circuit simulation of the ultrasound element (KLM model, see Methods 2.2) [18]. The measured frequency response was derived using the state-of-the-art sTIR characterization employing illuminated microsphere measurements (see Methods 2.2). The simulated frequency response suggests a peak amplitude at 3.4 MHz, the UT center frequency, and is approximately constant between 0 Hz and 500 kHz. On the other hand, the measured frequency response (blue) exhibits a peak amplitude at 500 kHz and only a local maximum at 3.4 MHz. The deviation for frequencies below 1 MHz can be attributed to the OptA signal generated by the microsphere, which is employed to measure the frequency response. The spectrum of the sphere signal, displayed in Fig. 1b (orange), exhibits very low signal amplitude in the range between 0 Hz and 1 MHz, with the amplitude decaying towards 0 at 0 Hz. Due to the low signal amplitude generated by the sphere at these low frequencies, the frequency response characterization becomes unreliable [14].

Opposed to the microsphere measurements, our proposed experimental arrangement utilizing reference Tx-UTs allows us to precisely probe the OptA tomography device at acoustic frequencies below 1 MHz. Fig. 1c showcases the mean and standard deviation (std) of the signal amplitude detected by a single Rx-UT in the center of the OptA array when 300 kHz (blue) and 500 kHz (orange) Tx-UTs were used as emitters. In comparison, Fig. 1c shows the average noise amplitude (black) between 25 kHz and 1 MHz and the OptA signal amplitudes generated by a microsphere at frequencies 500 kHz (pink) and 4 MHz (green) for reference. The mean amplitudes were computed over 100 pulses. The displayed signal amplitudes are not equal to the OptA imaging device’s frequency response, which is independent of the position of the emitter and Tx-UT electronics or OptA spectrum of a sphere, unless the SIR_x_ and the Tx-UT frequency response are deconvolved from the signal (see Methods 2.2). However, the SIR_x_ effect on the sub-1 MHz acoustic spectrum is negligible for a Tx-UT location in the far-field and a microsphere location in the array focus (see Supplementary Fig. A.2c,d). At 25 and 50 kHz, the mean signal amplitude detected by the Rx-UT from the Tx-UTs was approximately equal to the mean noise amplitude. At frequencies larger than 50 kHz, the mean signal amplitude detected from the Tx-UTs increased, while the noise decreased. At frequencies higher than 75 kHz, the mean signal received from the Tx-UTs significantly surpassed the noise. In measurements taken using the 500 kHz Tx-UT, we observed that the detected amplitude decayed symmetrically around the 500 kHz center frequency. Using the 300 kHz Tx-UT, we observed that the signal detected by the OptA Rx-UT still decayed around the 300 kHz center frequency, but without the apparent symmetry of the amplitude received from the 500 kHz Tx-UT. To infer the OptA imaging device’s frequency response, we divided the amplitude spectra by the Tx-UT frequency response to correct for the Tx-UT’s electronic behavior (see Methods 2.2). Following this step, the obtained acoustic spectrum was flat between 125 kHz and 300 kHz for the 300 kHz Tx-UT, while the amplitude detected from the 500 kHz Tx-UT declined over the same frequency range (see Supplementary Fig. A.1). For all collected acoustic spectra, the amplitude std across pulses was negligibly small for frequencies larger than 50 kHz. However, the amplitude std was significantly higher at 25 and 50 kHz (∼6 dB), similar to the noise std.

Besides the detection variability in a single Rx-UT for multiple pulses, there is also a variation between Rx-UTs in the OptA probe array. Fig. 1d shows the mean amplitude and standard deviation of the detected signal amplitude from a single pulse averaged across all Rx-UTs. Here, the amplitude means were lower than those averaged across pulses for both Tx-UTs (Fig. 1c), while the stds increased compared to the stds averaged across pulses. The stds across Rx-UTs were on average 4.49 dB and 5.37 dB larger than the stds across pulses for the 300 kHz and 500 kHz Tx-UTs, respectively. These changes were not observed in the noise spectrum or in the reference signal from the microsphere, both of which had means and stds similar to those plotted in Fig. 1c.

Fig. 1e,f depict the mean SNRs corresponding to the spectra plotted in Fig. 1c,d, respectively. Here, we observed very low mean SNRs of 1.17 dB at 25 kHz and 2.02 dB at 50 kHz for the 300 kHz Tx-UT averaged across pulses. The mean SNR of the 300 kHz Tx-UT at the same frequencies was even lower when averaged across Rx-UTs (-2.71 dB at 25 kHz and 0.82 dB at 50 kHz). Averaged across pulses, the mean SNR for the 300 kHz Tx-UT increased at 75 kHz to 15.85 dB and continued to increase towards 53.2 dB at 350 kHz, 6.22 dB higher than the SNR from the reference microsphere at 500 kHz and 0.25 dB lower than the SNR from the reference microsphere at 4 MHz. The mean SNR across pulses measured using the 500 kHz Tx-UT stayed nearly constant between 400 kHz and 750 kHz, remaining between the displayed SNRs of the microsphere signal. At larger frequencies, the SNR declined, but the decline was less prominent when divided by the Tx-UT frequency response (see Supplementary Figure A.1). Analogous to the mean amplitude across Rx-UTs, the mean SNR across Rx-UTs was lower than the mean SNR of a single Rx-UT across pulses for both Tx-UTs. The SNRs obtained from the microsphere measurements did not decrease. The stds of the SNRs also increased when averaged across Rx-UTs, similar to the stds of the signal amplitudes.

Our low-frequency SNR measurements reveal that an OptA tomography device can detect acoustic signals at frequencies as low as 75 kHz with sufficiently high amplitude to differentiate them from noise, i.e., an average SNR larger than 3 dB corresponding to a 2-fold higher signal than noise energy. Assuming 75 kHz to be the lower −3 dB cutoff frequency of the acoustic spectrum generated by an optical absorber upon illumination, the commercial OptA tomography device could be used to visualize a sphere with a diameter of up to 6.4 mm, a cylinder with a diameter up to 2.72 mm, or a layer up to 2.8 mm thickness without high pass filtering effects.

### 3.2 Visualizing low-frequency artifacts in simulated macroscopic OptA images

Fig. 2 demonstrates that reconstructing OptA images utilizing frequency responses with incorrect low-frequency signal amplitude, resulting from imprecise characterization measurements, can cause bulk artifacts in reconstructed OptA images. These artifacts are showcased in Fig. 2 using a numerical breast tissue phantom. Fig. 2a displays the true simulated frequency response of a commercial clinical OptA imaging device with limited bandwidth (FR, orange), alongside frequency responses with under- (FR_f,under_, green) and overestimated (FR_f,over_, pink) amplitude at frequencies below 1 MHz. Fig. 2b displays the numerical breast phantom at 930 nm.

The image reconstruction obtained with the true frequency response in Fig. 2c highlights that the second bulk tissue region (arrow 1) below the skin and the first lipid layer cannot be reconstructed precisely, and no contrast is observable in the reconstructed image. Equally, bulk contrast above the skin line (arrow 2) is not visible in the reconstructed image despite being present in the ground truth image. Fig. 2d depicts a depth profile from the center of the image (along the yellow dashed line in Fig. 2b), which confirms our qualitative observations. There is no contrast for multiple pixels above the skin line (point 2), and for depths beyond 100 pixels, the contrast drops significantly, showing strong fluctuation in the reconstructed image (point 1, orange solid line). In comparison, the profile line along the ground truth image (black dashed line) shows an exponential decay over depth with notable contrast up to 200 pixels.

The image reconstructed with a frequency response with underestimated low-frequency amplitude (FR_f,under_) in Fig. 2e shows a thin layer with enhanced contrast directly below the skin but much shorter than the true bulk tissue structure (arrow 1). The profile line in Fig. 2d (green solid line) shows an abrupt decline in contrast shortly after the skin line (point 1). Deeper in the tissue, an artificial layer emerges in Fig. 2b (arrow 3), which qualitatively has no similarity to any biological structures visible in the ground truth image, despite similar contrast (point 3). It does not appear in the image reconstructed with the true frequency response.

Reconstructing OptA images with a frequency response with overestimated low-frequency amplitude (FR_f,over_), as displayed in Fig. 2f, results in an artificially elongated bulk layer below the skin line with increased contrast (arrow 1) overlaying lower contrast structures. Besides the elongated bulk tissue layer below the skin, a high-contrast bulk tissue layer above the skin (arrow 2) exceeds the contrast in the ground truth image. The depth profile in Fig. 2d confirms the elongation of the bulk tissue layer (point 1) and the high-contrast bulk layer above the skin (arrow 2), unveiling an unrealistically strong signal at depths below 150 pixels.

The same artifacts caused by reconstructing OptA images with a frequency response with incorrect low-frequency amplitude which are visible the realistic breast tissue phantom, also appear in a numerical phantom displaying bulk tissue contrast only (three different layers, see Supplementary Fig. A.3). These observations suggest that low-frequency artifacts mainly appear in connection with bulk structures and less with finer tissue structures like vessels.

The qualitative observations of low-frequency artifacts in reconstructed OptA images are substantiated by the quantitative image quality assessment in terms of SSIM and MSE in Table 1. As expected, the reconstruction with true frequency response (recon FR) does not fully resemble the ground truth image achieving a SSIM below one and a MSE larger than zero. The reconstruction with a frequency response with underestimated low-frequency amplitude (recon FR_f,under_) has the lowest similarity with the ground truth image (lowest SSIM_GT_ and highest MSE_GT_ of all reconstructed images). However, the reconstruction with a frequency response with overestimated low-frequency amplitude (recon FR_f,over_) has the least similarity with the reconstruction with true frequency response (lowest SSIM_Recon,FR_, and highest MSE_Recon,FR_ of all reconstructions), which is expected in realistic macroscopic OptA imaging scenarios.

**Table 1:** Quantitative evaluation of low-frequency artifacts occurring in image reconstruction with imprecise frequency response characterization in sub-1 MHz frequencies. Evaluation of low-frequency image artifacts in terms of Structural Similarity Index (SSIM) and mean squared error (MSE) of the reconstructions with true frequency response (Recon FR, Fig. 2c), frequency response with underestimated low-frequency amplitude (Recon FR_f,under_Fig. 2e), and frequency response with overestimated low-frequency amplitude (Recon FR_f,over_Fig. 2f) in comparison to the ground truth (GT) and the reconstruction with true frequency response.

Recon type	Recon FR (Fig. 2c)	Recon FR_f,under_ (Fig. 2e)	Recon FR_f,over_ (Fig. 2f)
$\mathrm{SSI}M_{\mathrm{GT}}$	0.957	0.921	0.927
${\mathrm{MSE}}_{\mathrm{GT}}$	$1.605\;\times 10^{-5}$	$1.445\;\times 10^{-4}$	$7.996\;\times 10^{-5}$
$\mathrm{SSI}M_{\mathrm{Recon FR}}$	1	0.942	0.878
${\mathrm{MSE}}_{\mathrm{Recon FR}}$	0	$1.590\;\times 10^{-4}$	$1.080\;\times 10^{-4}$

Our results show that overestimating the low-frequency amplitude during reconstruction artificially extends bulk contrast layers, while underestimating the low-frequency amplitude artificially shortens bulk layers and introduces a deep, artificial bulk layer that may overlay real tissue contrast. Over- or underestimating the frequency response low-frequency amplitude reduced image fidelity in terms of SSIM and MSE, thus, may cause confusion or misinterpretation.

## 4 Discussion

In this work, we measured the lowest detectable frequency of a commercial, clinical macroscopic OptA imaging device for the first time, allowing us to accurately select the lower cut-off frequency of the signal filter applied in OptA image reconstruction. Correctly setting this threshold would enable optimal noise filtering while preserving the maximum amount of signal information. Moreover, these measurements allowed us to estimate the maximum scale at which bulk tissue can be visualized in OptA images without high pass filtering effects, despite the bandwidth limitation of the UT employed for signal detection. This allows operators to better judge the capabilities of OptA imaging devices and enables them to select the correct cutoff frequencies for the bandpass signal filter in image reconstruction to achieve maximum image fidelity. This is particularly important as bulk tissue structures have stronger contrast than fine structures in OptA images [7] and provide functional and molecular contrast, which carries important clinical information [9]. We additionally investigated low-frequency image artifacts, which are triggered by reconstructing images utilizing a frequency response with over- or underestimated amplitude for frequencies smaller than 1 MHz in simulations. These investigations serve as a reference for the size and appearance of bulk tissue artifacts in reconstructed OptA images, assisting clinicians and researchers in identifying low-frequency artifacts.

We observed that the state-of-the-art frequency response characterization method, which uses laser-illuminated microspheres, was not able to generate sufficient signal amplitude to reliably probe the sub-1 MHz sensitivity of a clinical OptA imaging device. This agrees with previous findings, where laser-illuminated slabs [14] or thin plates [35] have been utilized instead of microspheres for frequency response characterization due to their flatter spectra and higher signal at low frequencies. However, the low-frequency Tx-UTs used in our experiment are optimized for signal transmission at frequencies below 1 MHz and provide higher signal quality at these frequencies than slabs, thin plates, or microspheres. This improved quality can be seen, for example, in a comparison of the frequency response of the microsphere (see Fig. 1b) and the Tx-UTs (see Supplementary Fig. A.1a). Hence, low-frequency Tx-UTs allow us to measure the low-frequency SNR reliably. Through our measurements, we prove that our commercial OptA imaging device can detect signals as low as 75 kHz with an average SNR larger than 3 dB, enabling reliable differentiation of signal and noise. We demonstrate signal detection at frequencies far beyond the simulated 3 dB and 6 dB cutoff frequencies of 2.4 MHz and 1.5 MHz, respectively, albeit with much lower sensitivity. Hence, bulk structures up to an approximate diameter of 6.4 mm (sphere) or 2.72 mm (cylinder) or 2.8 mm thickness (layer) can be visualized using our OptA imaging device without high-pass filter effects, as previously investigated [8], if the lower cutoff frequency of the bandpass filter in image reconstruction is selected correctly.

However, the amplitude and SNR spectra measured with our proposed technique do not resemble the frequency response of the OptA imaging device. The frequency response of the OptA imaging device must be independent of the acoustic emitter, which can be achieved by deconvolution of the Tx-UTs’ frequency response. However, performing this correction by deconvolving the square root of the Tx-UTs’ pulse-echo frequency response characterization from the measured signals suggested SNRs larger than 10 dB for frequencies below 50 kHz (see Supplementary Fig. A.1b-d). However, a physical high-pass filter at 50 kHz, implemented in the device’s detection electronics, inhibits these detection SNRs for frequencies below 50 kHz, suggesting an imprecise Tx-UT frequency response characterization. This inaccuracy may be caused by the narrow-band filtering applied during the pulse-echo characterization of the Tx-UTs. It may be addressed by calibrating the Tx-UTs’ frequency responses with dedicated hydrophones in the future.

Moreover, the increased variance in amplitude and SNR across Rx-UTs compared to the variance across pulses for the Tx-UTs (see Fig. 1c-d) contradicts the SIR_x_ simulations, suggesting that the location of the Tx-UT has negligible influence on detected signal amplitude and thus also SNR for acoustic sources in the far-field (see Supplementary Fig. A.2a,b). This suggests reflection of the acoustic waves on the transducer surface due to acoustic impedance mismatch, which could be accounted for in the future with precise modeling of the UTs’ acoustic material properties or angle-dependent measurements of the detected signal amplitude.

While our measurements allow us to set the filter cutoff frequency in signal processing to maximize image fidelity, the low-frequency amplitude of the device’s frequency remains unknown. Therefore, as investigated here, low-frequency artifacts remain likely to occur in macroscopic OptA images and may be misjudged for factual tissue contrast, potentially leading to wrong clinical decisions. It was expected that only the entry layer and not the entire bulk tissue would be observable in an OptA image, i.e., spatial high-pass filtering [8], because the frequency response of a commercial clinical OptA imaging device with limited bandwidth was used in this experiment. However, reconstruction using a frequency response with overestimated low-frequency amplitude artificially elongated bulk layers, while reconstruction using a frequency response with underestimated low-frequency amplitude introduced additional bulk layer artifacts deep in tissue, which may obscure factual tissue contrast (see Fig. 2, Table 1, and Supplementary Fig. A.3).

To the best of our knowledge, this is the first assessment of low-frequency artifacts in reconstructed OptA images. Previous studies have shown compelling image resolution and contrast enhancement using Total Impulse Response correction in OptA microscopy [36] and tomography [12], [13], [37]. In contrast, other studies have investigated artifacts introduced by device properties like streaks due to limited view detection [38], [39] or the previously mentioned spatial high pass filtering due to limited detection bandwidth [8]. However, no investigation of imprecise sub-1 MHz frequency response characterization has been conducted. Our results are especially important because state-of-the-art UT frequency response characterization techniques employing illuminated microspheres are error-prone for frequencies below 1 MHz; hence, low-frequency artifacts are likely to occur [14].

Such artifacts can only be mitigated by improving the isTIR method [13], [23] with a precise sub-1 MHz frequency response. In the future, we propose integrating a SIR_x_ model that accounts for reflections on the UT’s surface. Moreover, a set of hydrophone-calibrated reference Tx-UTs with overlapping emission spectra should be employed as acoustic sources, allowing the characterization of the detectors’ frequency response with high SNR for sub-1 MHz frequencies. The obtained physical model of the imaging device and process will have higher fidelity than the current isTIR models obtained with laser-illuminated microspheres, resulting directly in higher image fidelity when employed in model-based reconstruction [40], [41].

Nevertheless, we have only investigated artifacts in two cases: a tissue-mimicking breast tissue phantom and a numerical phantom with three layers mimicking layered bulk tissues. Human tissue varies for different body locations, meaning that noise, as well as fluence decay, will affect the image appearance notably. Hence, artifacts will be much harder to identify in a different clinical setting. An extended study with multiple numerical phantoms mimicking different tissue shapes should be conducted in the future to provide more insight. Generally, the results would be most compelling if they could be presented in physical phantoms or in vivo human data. However, as it is so far impossible to accurately characterize the frequency response of an OptA tomography device over the full bandwidth, this poses an impossible challenge today.

Our measurements allow us to determine that a clinical OptA imaging device can detect acoustic signals at frequencies far lower than its simulated bandwidth, or than the bandwidth derived from measurements with laser illuminated microspheres, may suggest, which allows accurate representation of bulk tissue structures up to a scale of several millimeters. However, our measurements did not measure the device’s frequency response across the entire bandwidth of the detection system of the clinical OptA device. Consequently, our measurements do not provide a quantitative comparison between a device’s sub-1 MHz sensitivity and its peak sensitivity at higher frequencies. Thus, we can not infer if such signal amplitudes could be achieved with safe laser energy levels in human or animal operation. In the future, these limitations should be addressed by using a set of calibrated Tx-UTs with overlapping frequency response to cover the entire frequency band of an OptA imaging device’s UTs and acquisition electronics, thus quantitatively characterizing the device’s full frequency response and the detection limit in terms of pressure amplitude providing insights into the necessary laser energy levels to generate the signal amplitudes for detection. These results should also be compared with frequency characterization measurements employing thin plates or slabs to establish their accuracy at sub-1 MHz frequencies. Equally, bulk tissue artifacts caused by inaccurate frequency response characterization could be investigated in physical phantoms and *in vivo* tissue.

Our work shows that it is possible to investigate bulk tissue with macroscopic OptA imaging devices, which are already in use in clinical settings. Hence, general morphology as well as functional and molecular parameters can be derived from bulk tissue in OptA images, which can potentially aid the detection and monitoring of treatment of diseases such as breast cancer or peripheral neuropathy.

## CRediT authorship contribution statement

**Maximilian Bader:** Writing – review & editing, Writing – original draft, Visualization, Validation, Project administration, Methodology, Investigation, Formal analysis, Conceptualization. **Antonia Longo:** Writing – review & editing, Resources, Methodology, Investigation, Conceptualization. **Dominik Jüstel:** Writing – review & editing, Validation, Supervision, Resources, Project administration, Funding acquisition, Conceptualization. **Vasilis Ntziachristos:** Writing – review & editing, Validation, Supervision, Resources, Project administration, Funding acquisition, Conceptualization.

## Funding

This project has received funding from the European Research Council (10.13039/100010663ERC) under the European Union’s Horizon 2020 research and innovation programme under grant agreement No 694968 (PREMSOT) and under Horizon Europe under grant agreement No 101041936 (EchoLux), from the Deutsche Forschungsgemeinschaft (10.13039/100004807DFG) - 455422993 as part of the Research Unit FOR 5298 (iMAGO, subproject TP2, GZ: NT 3/32–1) and from the Bavarian Ministry of Economic Affairs, Energy and Technology (StMWi) (DIE-2106–0005// DIE0161/02, DeepOpus).

## Declaration of Competing Interest

The authors declare the following financial interests/personal relationships which may be considered as potential competing interests: Antonia Longo reports equipment, drugs, or supplies was provided by iThera Medical GmbH. Antonia Longo reports a relationship with iThera Medical GmbH that includes: employment. Vasilis Ntziachristos reports a relationship with Biosense Innovations P.C.that includes: equity or stocks. Vasilis Ntziachristos reports a relationship with Maurus OY that includes: equity or stocks. Vasilis Ntziachristos reports a relationship with sThesis GmbH that includes: equity or stocks. Vasilis Ntziachristos reports a relationship with Spear UG that includes: equity or stocks. Vasilis Ntziachristos reports a relationship with I3 Inc that includes: equity or stocks. Antonia Longo has patent #EP22177153.8 and PCT/EP2023/064714 pending to iThera Medical GmbH and Helmholtz Zentrum München GmbH. Dominik Jüstel has patent #EP22177153.8 and PCT/EP2023/064714 pending to iThera Medical GmbH and Helmholtz Zentrum München GmbH. If there are other authors, they declare that they have no known competing financial interests or personal relationships that could have appeared to influence the work reported in this paper.

## Data Availability

Data will be made available on request.

## Footnotes

Appendix A Supplementary data associated with this article can be found in the online version at doi:10.1016/j.pacs.2025.100785.
