# Supplementary material for: Low frequency detection in clinical multispectral optoacoustic tomography
Source: Photoacoustics. 2025 Nov 17;46:100785. doi: 10.1016/j.pacs.2025.100785 (PMC12677182; doi:10.1016/j.pacs.2025.100785)
Supplement: Supplementary file 1 — Supplementary material [file mmc1.docx]

**Low Frequency Detection in Clinical Multispectral Optoacoustic Tomography**

Maximilian Bader^a,b^, Antonia Longo^b,c^, Dominik Jüstel^a,b.d^, Vasilis Ntziachristos^a,b,e,f,^[^✉^](https://www.google.com/url?sa=t&rct=j&q=&esrc=s&source=web&cd=&ved=2ahUKEwimwNbn9LH3AhWnQ_EDHcVTCXIQFnoECAUQAQ&url=https%3A%2F%2Fwww.compart.com%2Fen%2Funicode%2FU%2B2709&usg=AOvVaw3wH6-qM59agxmnZvvhCFIa)

^a^ Chair of Biological Imaging, Central Institute for Translational Cancer Research (TranslaTUM), School of Medicine and Health & School of Computation, Information and Technology, Technical University of Munich, Munich, Germany

^b^ Institute of Biological and Medical Imaging, Bioengineering Center, Helmholtz Zentrum München, Neuherberg, Germany

^c^ iThera Medical GmbH, Munich, Germany

^d^ Institute of Computational Biology, Computational Health Center, Helmholtz Zentrum München, Neuherberg, Germany

^e^ Institute of Electronic Structure and Laser (IESL), Foundation for Research and Technology Hellas (FORTH), Heraklion, Greece

^f^ Munich Institute of Biomedical Engineering (MIBE), Technical University of Munich, Garching b. München, Germany

[^✉^](https://www.google.com/url?sa=t&rct=j&q=&esrc=s&source=web&cd=&ved=2ahUKEwimwNbn9LH3AhWnQ_EDHcVTCXIQFnoECAUQAQ&url=https%3A%2F%2Fwww.compart.com%2Fen%2Funicode%2FU%2B2709&usg=AOvVaw3wH6-qM59agxmnZvvhCFIa) Corresponding author. Email: bioimaging.translatum@tum.de, Address: Chair of Biological Imaging, Technical University of Munich, Ismaninger Straße 22, D-81675 Munich, Germany

**A. Supplementary Information**

**A.1. Low-Frequency Signal Amplitude and SNR Measurements Corrected for the Reference Transducer Frequency Response**

Fig. A.1 illustrates that the amplitude and SNR spectra of a clinical OptA imaging device in the frequency range of 25 kHz to 1 MHz appear corrupted after correction for the square root of the pulse-echo frequency response of the Tx-UT. Fig. A.1a displays the square-root frequency response of the 300 kHz (blue) and the 500 kHz Tx-UT (orange) derived from the pulse-echo characterization. The mean amplitude spectrum across pulses corrected for the Tx-UT frequency response is plotted in Fig. A.1b and suggests a higher signal than noise at 25 kHz and 50 kHz. However, when the noise is equally corrected for the frequency response of the 300kHz TX UT, noise and signal amplitude become approximately equal again. Signal only becomes significantly higher than noise independent of the correction with the Tx-UT frequency response at frequencies larger than 75 kHz.

For frequencies larger than 100 kHz, the signal amplitude spectrum of the 300 kHz Tx-UT stays flat, while the signal amplitude spectrum of the 500 kHz Tx-UT decays. Following eq. (2) (see Methods 2.1), the amplitude spectrum should be equal for both Tx-UTs, as the spectrum should only be dependent on the OptA imaging device. In Fig. A.1c, the mean and std of the amplitude spectrum across Rx-UTs show the same reduction in mean amplitude and increase in variance as the amplitude spectra without Tx-UT frequency response correction in Fig. 1d. The SNR mean and std across pulses and across Rx-UTs, displayed in Fig. A.1d,e respectively, show an SNR larger than 0 dB for 25 kHz and 50 kHz, which suggests that the OptA imaging device is able to record signal at these frequencies. However, a high-pass signal filter integrated into the hardware of the OptA imaging device filters these low acoustic frequencies, so signal detection is not feasible. Moreover, the noise amplitude corrected for the Tx-UT frequency response showed an amplitude with similar mean and variance (see Fig. A.1b). Similar to the amplitude spectra in Fig. A.1b,c, we observe a rise in SNR with varying slope for the 300 kHz and the 500 kHz Tx-UTs for frequencies between 100 kHz and 400 kHz. Ultimately, correcting for the square-root pulse-echo frequency response of the Tx-UTs results in amplitude and SNR spectra which vary depending on the Tx-UT even though they should be equal except for a scalar. Also, the corrected SNR spectra suggest that the OptA imaging device can detect acoustic frequencies at 25 kHz and 50 kHz but is inhibited by the acquisition electronics' hardware.

**
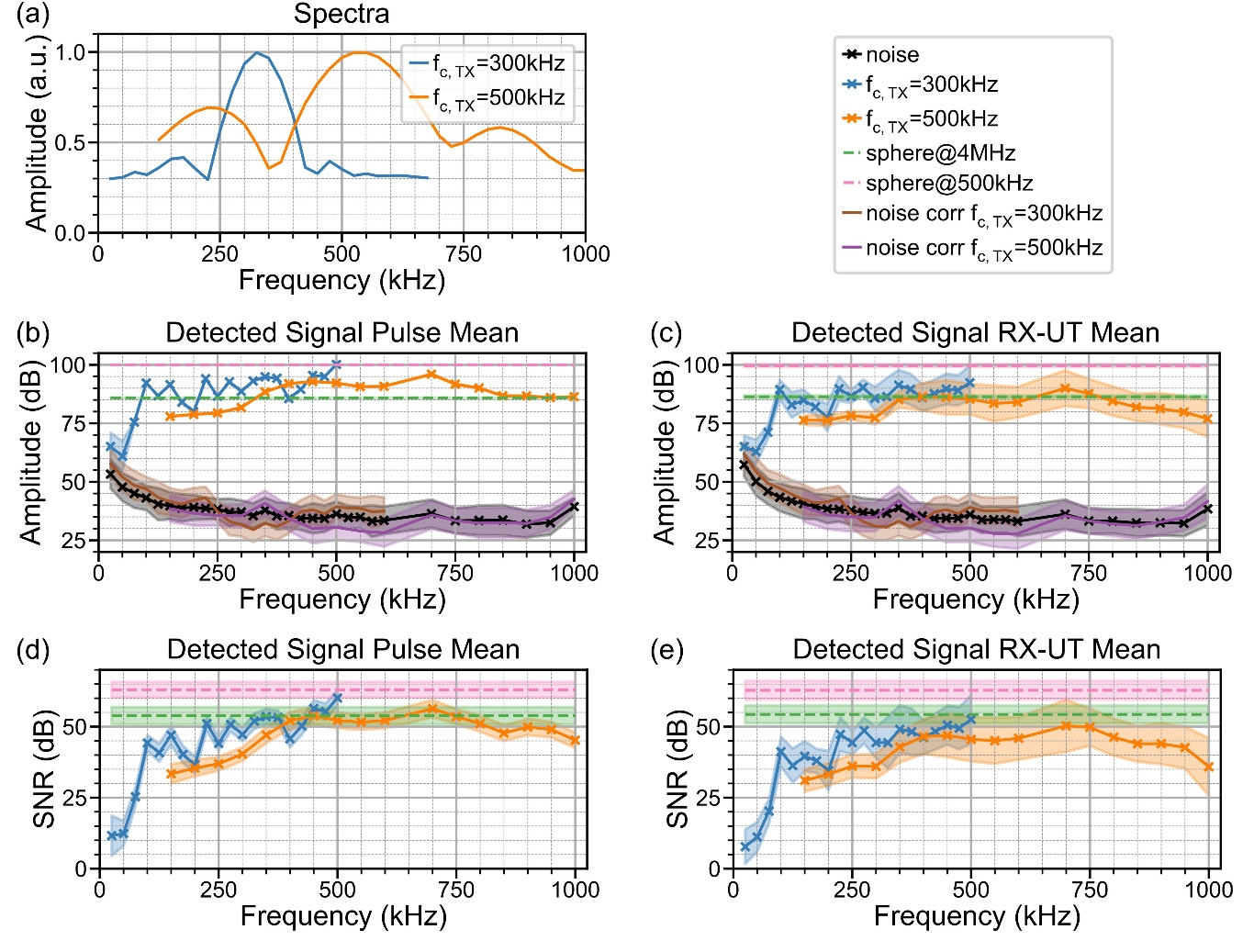
**

**Figure A.1: Measured low-frequency signal-to-noise ratio (SNR) of a clinical macroscopic optoacoustic (OptA) imaging device for frequencies between 25 kHz and 1 MHz without correcting for the transmitting transducer’s (Tx-UTs) frequency response (FR).** (a) Frequency responses of the reference transducers with 300 kHz center frequency ($f_{c,TX}=300 kHz$, blue) and with 500 kHz center frequency ($f_{c,TX}=500 kHz$, orange) derived from the pulse-echo characterization performed by the manufacturer. (b,c) Mean and standard deviation (std) of the signal amplitude across a frequency range from 25 kHz to 1 MHz, for a single receiving ultrasound transducer (RX-UT) averaged over 100 pulses (c) and for a single pulse averaged over 256 RX-UTs (d) for Tx-UTs with center frequencies of 300 kHz (blue) and 500 kHz (orange). Raw noise is shown in black, noise corrected for the $f_{c,TX}=300kHz$ Tx-UT electrical impulse response (EIR) is shown in brown and noise corrected for the $f_{c,TX}=500kHz$ Tx-UT EIR is shown in purple. For comparison, dashed lines show that the optoacoustic (OptA) signal amplitude generated by a microsphere at frequency $f=4 MHz$ (green) and at $f=500 kHz$ (pink). (d,e) Mean and std of the SNR for a single RX-UT averaged over 100 pulses (e) and for a single pulse averaged over 256 RX-UTs (f) for reference transducers with center frequencies of 300 kHz (blue) and 500 kHz (orange). Dashed lines show the SNRs of a microsphere at $f=4 MHz$ (green) and at $f=500 kHz$ (pink).

**A.2. Effect of Reference Transducer Location on Recorded Amplitude Spectrum**

Fig. A.2a-b display the amplitude spectrum of the spatial impulse response (SIR_x_) for the 256 transducers in the optoacoustic (OptA) imaging device’s probe array (see Methods 2.2 for array specifications) for a virtual point source located at the focus of the reference transmission ultrasound transducers (Tx-UTs) with center frequencies of 300 kHz and 500 kHz, respectively. Due to the varying focus lengths of the two TX UTs, the exact location of the virtual TX varied, but both locations still resided in the far-field of all receiving ultrasound transducers (Rx-UTs) in the array. For both Tx-UT locations, the spectrum relative to the amplitude at $f=0 Hz$ (DC) shows that there is only minimal decay up to a frequency of 1 MHz. Moreover, the amplitude variation for different Rx-UTs in the array is negligibly small despite their orientation and location. Because the decay and the variation of the Rx-UT position and orientation are negligibly small in the frequency range between 0 and 1 MHz, the SIR_x_ does not have to be accounted for in the amplitude spectrum and signal-to-noise ratio (SNR) measurements.

Fig. A.2c showcases the attenuation of acoustic waves in water (speed of sound $c=1500 m/s$, $\alpha_{0,f}=0.00217 dBMHz^{-1}cm^{-1}$, $n=2$). The attenuation was modeled using an exponential law for the distance between the center Rx-UT in the array and either the microsphere location or the virtual focus of the 300kHz and 500kHz Tx-UTs [1]. Fig. A.2d displays the frequency response of the corresponding filter kernel, encapsulating attenuation and dispersion effects [2]. Fig. A.2c shows that the attenuation in water is 0 dB and correspondingly the filter kernel amplitude is equal to 1. Hence, attenuation and dispersion, like the SIR_x_, are negligible for measurements in water and do not have to be accounted for in the amplitude spectrum and SNR measurements.


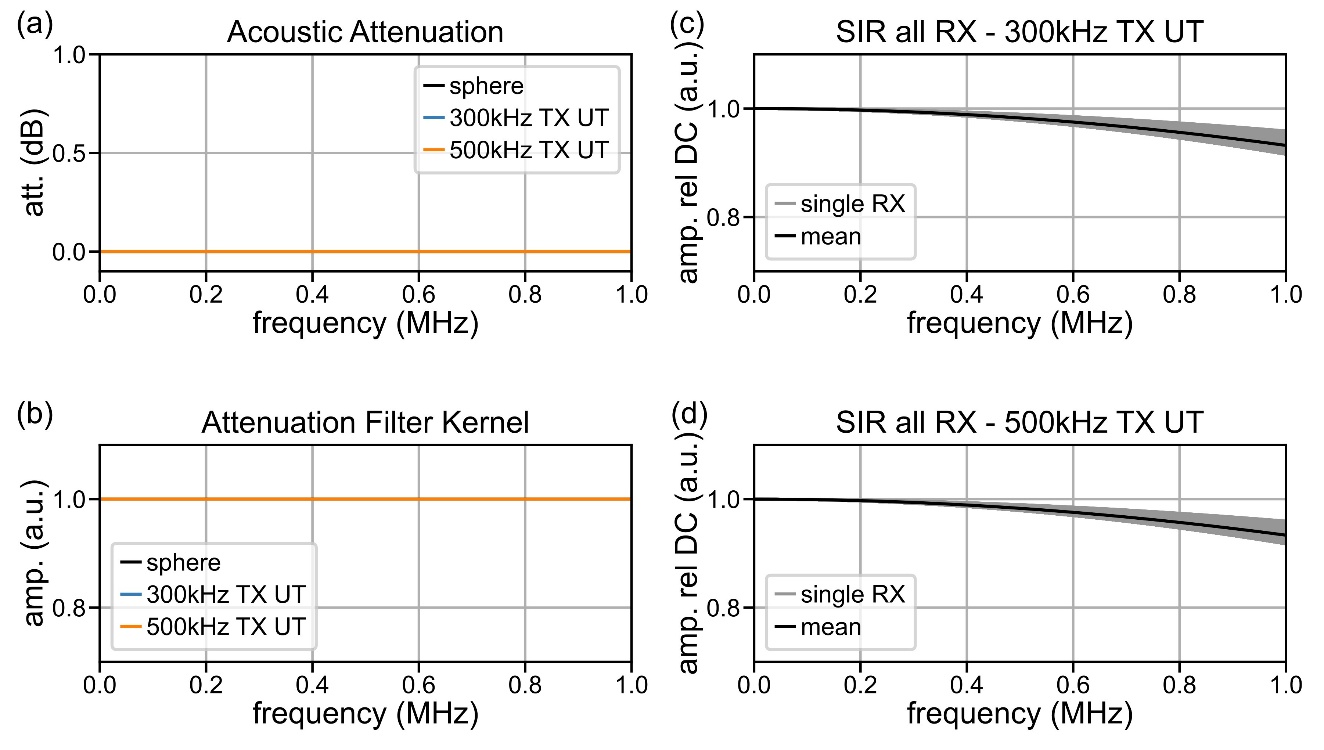


**Figure A.2: Effect of spatial impulse response (SIR) and attenuation on measurements of the low-frequency signal-to-noise ratio (SNR) of a clinical macroscopic optoacoustic (OptA) imaging device.** (a,b) The relative SIR amplitude spectrum for 256 receiving ultrasound transducers (RX-UTs) in the OptA probe array and the mean amplitude over all RX,-UTs given transmission ultrasound transducers (Tx-UTs) with $f_{c,TX}=300 kHz$ (spherical focus radius: 6 mm; a) and $f_{c,TX}=500 kHz$ (spherical focus radius: 4 mm; b) at the virtual source location between 0 Hz and 1 MHz relative to the amplitude at $f=0 Hz$ (DC). (c,d) Attenuation (c) and attenuation filter kernel (d) for acoustic waves in water over the distance between the microsphere, the Tx-UT with $f_{c,TX}=300 kHz$ and the Tx-UT with $f_{c,TX}=500 kHz$ in a frequency range between 0 Hz and 1 MHz. amp. rel. DC – amplitude relative to DC, att. – attenuation, amp. – amplitude, dB - decibel.

**A.3. Visualizing Low-frequency Artifacts in A Simulated Macroscopic Layer Tissue Phantom**

In addition to the numerical breast tissue phantom (see Fig. 2), we also investigated the effects in a numerical layer phantom consisting of layers to investigate the low-frequency artifacts in bulk structures only. Fig. A.3a displays the frequency response with true, over- and underestimated low-frequency amplitude which were employed for image reconstruction (equal to frequency responses displayed in Fig. 2a). Fig. A.3b depicts the numerical phantom, which consisted of three layers with exponentially decaying initial pressure. In the layers, the initial pressure was defined by a power law:

| $p_{0}^{i}\left( x,z \right)=\mu_{1,i}\exp\left( -\mu_{2,i}\left( z-z_{s,i} \right) \right), z_{s,i}\leq z\leq z_{e,i},$ | $(9)$ |
| --- | --- |

where $i\in\{1,2,3\}$ denotes the layer number, $z_{s,i}$ denotes the starting depth and $z_{e,i}$ denotes the ending depth of the layer. $\mu_{1,i}$ serves as the initial pressure constant and $\mu_{2,i}$ as the effective extinction coefficient (first layer: $z_{s,1}=-9 mm, z_{e,1}=-8 mm, \mu_{1,1}=10, \mu_{2,1}=10$, second layer: $z_{s,2}=-8 mm, z_{e,2}=-5 mm, \mu_{1,2}=5, \mu_{2,2}=2$, third layer: $z_{s,3}=-5 mm, z_{e,3}=10 mm, \mu_{1,3}=25, \mu_{2,3}=10$). Throughout the phantom, we defined a constant speed of sound $c=1510 m/s$.

Fig. A.3c shows an image of the numerical phantom reconstructed with the true frequency response. Here, we observed that only the entry layer into the bulk tissue was visible, indicating that the bulk structure is spatially high pass filtered (arrow 1). Additionally, we observed a layer deep below the last bulk layer (arrow 2). Fig. A.3d depicts a depth profile from the center of the image (along the yellow dashed line in Fig. A.3b) which confirms our qualitative observations. The deviation in bulk contrast (point 1) is clearly visible in the reconstruction with true frequency response (Recon FR; orange line), compared to the ground truth image (black dashed line). The emergence of a layer after the last factual layer (point 2) is also visible.

Fig. A.3e displays the image reconstructed with a frequency response that underestimates the low-frequency amplitude (FR_f,under_, green in Fig. A.3a). In this image, we observed greatly shortened entry layers of the bulk layers (arrow 1) compared to both the ground truth and the reconstruction with the true frequency response. Moreover, the bulk layer artifact below the last layer was clearly visible and had increased contrast compared to the reconstruction with the true frequency response. This enhancement can also be observed in Fig. A.3d (green profile line).

Fig. A.3f depicts the image reconstructed with an overestimated low-frequency amplitude (FR_f,over_, pink in Fig. A.3a). In this image, we observed artificially elongated bulk layers (arrow 1) and an enhanced bulk layer artifact above the first layer interface (arrow 3) compared to the reconstruction with true frequency response. The profile line in Fig. A.3d (pink) shows that the bulk layer (point 1) is artificially enhanced. The depth profile through this layer looks similar to the ground truth, except for a section of zero-contrast before the next layer and the strong layer artifact above the first interface.

In terms of quantitative image metrics displayed in Table A.1, we observe that the reconstruction with frequency response with true low-frequency amplitude (recon FR) has lowest similarity with the ground truth image in terms of SSIM. Like for the numerical breast tissue phantom, the reconstruction with frequency response with underestimated low-frequency amplitude (recon FR_f,under_) is less similar to the ground truth image than the reconstruction with frequency response with overestimated low-frequency amplitude (recon FR_f,over_) resulting in a lower SSIM_GT_ and higher MSE_GT_. On the other hand, the reconstruction with underestimated low-frequency amplitude is more similar to the image reconstructed with true frequency response in terms of SSIM_recon,FR_ and MSE_recon,FR_ than the reconstruction with overestimated low-frequency amplitude.

Overall, the low-frequency artifacts observed in the simplistic three-layer phantom correspond to the observations in the realistic breast tissue phantom (see Fig. 2). The low-frequency artifacts observed, artificially shortened or elongated bulk tissue layers and the emergence of non-physical bulk tissue layers in depth, diminish overall image quality but mainly affect bulk tissue contrast fidelity which inhibits the reliable interpretation and inference of functional tissue parameters.

**
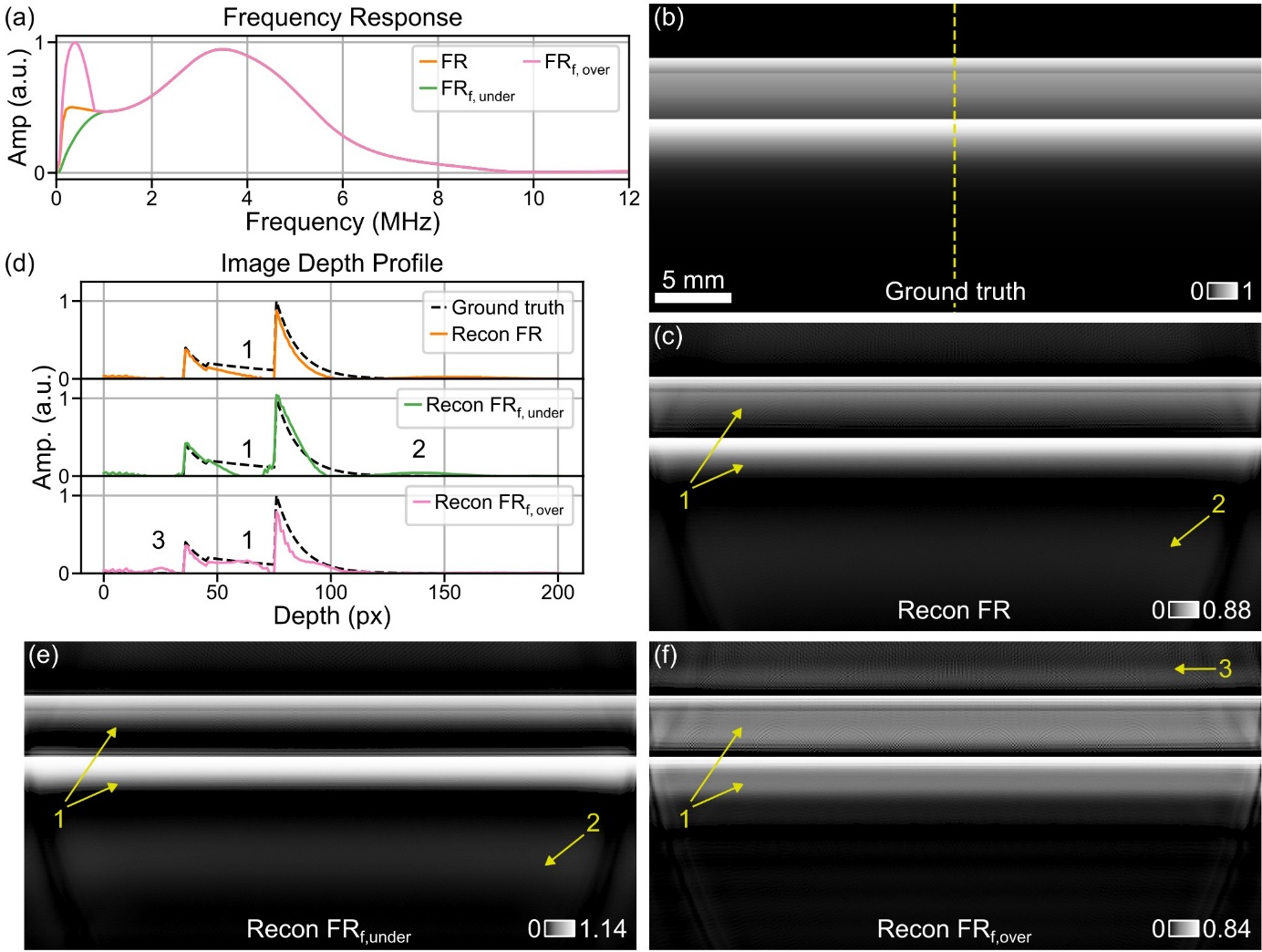
**

**Figure A.3: Numerical phantom experiment to qualitatively investigate the effects of imprecise transducer frequency response characterization for sub-1 MHz frequencies.** (a) Simulated frequency response (FR) of a clinical macroscopic optoacoustic (OptA) imaging device with true (orange), over- (pink) and underestimated (green) amplitude for low frequencies (<1 MHz). (b) A numerical phantom representing bulk tissue. The yellow dashed line indicates the location of the image depth profiles displayed in (b) and the numbers correspond to the numbers displayed in (b,c,e,f). (c) An image of the bulk tissue phantom reconstructed with the true frequency response shows only an entry layer (arrow 1) instead of the entire bulk, and a low contrast bulk layer below the final layer (arrow 2). (d) Depth profiles across all images (b,c,e,f) at the same lateral position indicated by the yellow dashed line in (b). (e) Image of the numerical phantom reconstructed with a frequency response which underestimates the low-frequency amplitude (FR_f,under_) with significantly shortened entry layer (arrow 1) and an layer below the final layer (arrow 2). (f) Image of the numerical phantom reconstructed with a frequency response overestimating the low-frequency amplitude (FR_f,over_) showing artificially enhanced layers (arrow 1) and a layer above the first bulk layer (arrow 3). Abbreviations: Amp. – amplitude, px – pixel.

| Recon type | Recon FR (Fig. A.3c) | Recon FR_f,under_ (Fig. A.3e) | Recon FR_f,over_ (Fig. A.3f) |
| --- | --- | --- | --- |
| $SSIM_{GT}$ | $0.625$ | $0.692$ | $0.702$ |
| ${MSE}_{GT}$ | $1.599 \times{10}^{-3}$ | $1.851 \times{10}^{-3}$ | $2.254 \times{10}^{-3}$ |
| $SSIM_{Recon FR}$ | $1$ | $0.849$ | $0.761$ |
| ${MSE}_{Recon FR}$ | $0$ | $2.426 \times{10}^{-3}$ | $1.044 \times{10}^{-3}$ |

**Table A.1: Quantitative evaluation of low-frequency artifacts occurring in image reconstruction with imprecise frequency response characterization in sub-1 MHz frequencies.** Evaluation of low-frequency image artifacts in terms of Structucal Similarity Index (SSIM) and mean squared error (MSE) of the reconstructions with true frequency response (Recon FR, Fig. A.3c), frequency response with underestimated low-frequency amplitude (Recon FR_f,under_ Fig. A.3e), and frequency response with overestimated low-frequency amplitude (Recon FR_f,over_ Fig. 2f) in comparison to the ground truth (GT) and the reconstruction with true frequency response.

**References**

[1] T.L. Szabo, Attenuation, in: Diagnostic Ultrasound Imaging: Inside Out, 2014: pp. 81–119. https://doi.org/10.1016/B978-0-12-396487-8.00004-5.

[2] X.L. Deán-Ben, D. Razansky, V. Ntziachristos, The effects of acoustic attenuation in optoacoustic signals, Phys Med Biol 56 (2011) 6129–6148. https://doi.org/10.1088/0031-9155/56/18/021.
